# Integrating the Public Health Services Model into Age-Friendly Pharmacies: A Case Study on the Pharmacies in Taiwan

**DOI:** 10.3390/healthcare9111589

**Published:** 2021-11-19

**Authors:** Shih-Chang Chen, Kuan-Han Lee, Der-Juinn Horng, Po-Jui Huang

**Affiliations:** 1 Department of Business Administration, National Central University, Taoyuan 32001, Taiwan; chen591124@gmail.com; 2 Department of Pharmacy, Chia Nan University of Pharmacy and Science, Tainan 71710, Taiwan; kuanhanlee@mail.cnu.edu.tw; 3 Department of Medicinal and Applied Chemistry, Kaohsiung Medical University, Kaohsiung 80708, Taiwan

**Keywords:** age-friendly pharmacy, pharmaceutical service, community pharmacy, medication adherence, public health service

## Abstract

Taiwan is expected to become a superaged society by 2026. Community pharmacies have recently joined Taiwan’s primary care system; they have great potential to provide professional healthcare services. This study examined whether the services provided by community pharmacists enhance medication adherence, enable the identification and solution of drug therapy problems, and are accepted by community residents. The Department of Public Health, Taoyuan City, collaborated with the Taoyuan Pharmacist Association over 11 months in 2018 in enabling pharmacists to dispense prescriptions and provide medication adherence consultations, cognitive services, and home and institutional medical care services. This study designed four satisfaction questionnaires to assess the feasibility and performance of these services. Regarding the services related to medication knowledge and adherence, 92.10% of the patients reported overall satisfaction, and all understanding and ability scores were improved in more than 95% of patients. The number of patients highly cooperative regarding their medication had risen from 14 to 234 after the intervention, and the number with low medication adherence had dropped from 533 to 33. More than 90% of respondents indicated that the institutional medical care services had significantly improved their medication knowledge and behaviors. The feasibility of the incorporation of integrated the public health services model into age-friendly pharmacies was confirmed by this study.

## 1. Introduction

The 2007 Global Age-Friendly City Index compiled by the World Health Organization (WHO) indicates that the world’s population is aging. The percentage of people aged 60 years or older is expected to increase from 11% in 2006 to 22% in 2025. Taiwan became an aging society in 1993 and progressed to an aged society in 2018; the country is expected to become a superaged by 2026 [1]. According to the actuarial report made by the National Health Insurance (NHI) Administration in 2011, because the medical expense associated with each older adult is two to four times the national average medical expense, the financial burden of the NHI is expected to rise sharply. Yet, the increase in medical resource usage by people aged 65–79 years was found to be insubstantial. The changes in the patterns of healthcare due to population aging are the primary cause of changes in medical resource consumption [2]. Studies conducted worldwide have revealed that such changes will significantly increase the demand for healthcare and medical costs because medical care and usage costs for older adults are disproportionally high [3].

Population aging has affected healthcare systems. In Taiwan, 60% of older adults have received a diagnosis of two or more chronic diseases [4,5]. Polypharmacy is prevalent among these older adults. Moreover, repeat medical treatment has been reported because of older adults’ desire to cure their chronic diseases and their disappointment with the effectiveness of long-term medication [5]. Poor medication adherence, especially when patients with multiple chronic diseases take multiple medications and the timing of the medications is complicated, numerous drug therapy problems (DTPs) are inevitable. Studies have indicated that poor medication adherence leads to poor disease control by patients and may lead to rehospitalization or repeated emergency department visits; in extreme cases, early death can occur. Overall, 30–50% of all patients analyzed in these studies failed to contain their diseases because of poor medication adherence; 33–69% of patients were hospitalized as a result. Each year, approximately 120,000 deaths are strongly correlated with poor medication adherence. The consequent medical expense reaches USD 100–300 billion per year [6].

Community pharmacies in Taiwan have joined the primary care system; however, the provision of professional and sustainable services from community pharmacies and in coordination with primary medical teams still requires systematic integration. In an aging society, poor medication adherence requires pharmacists to provide pharmaceutical care. In response to the healthcare demand caused by a change in Taiwan’s population structure, the government gave pharmacists the opportunity to provide professional services to assist patients in acquiring safe, effective medication and medical resources. The Taiwanese government began enforcing the separation of prescribing and dispensing in 1999. Various pharmaceutical services have been offered to promote the transformation of community pharmacy services, and age-friendly environments were established in community pharmacies by 2015. This has led to the integration of pharmaceutical services, in which the medication history data can be downloaded from the NHI cloud system and perform an integrated evaluation. In 2017, 15 community pharmacies were selected in and promoted community-based integrated pharmaceutical services. On average, each pharmacist identified one or two DTPs for each patient serviced, including prescription-related problems. The medication adherence of patients improved, the patients were given appropriate medication knowledge, and institutional pharmaceutical care and referral services between hospitals and community pharmacies were executed [7].

In 2017, 65-year-old or older adults in Taoyuan City constituted more than 11.40% of the city’s total population [8]. More than 500 NHI-contracted pharmacies had been established by that year. Based on the concept of aging in place, residents are provided with local, accessible, and affordable pharmaceutical services to promote self-care, correct medical, and medication behaviors, thereby improving patients’ life quality. Therefore, constructing the age-friendly pharmacies integrated with public health services to satisfy the unmet needs in Taoyuan City is critical.

The objective of this study is to construct age-friendly community pharmacies integrated with the public healthcare services. Additionally, this study will examine whether the professional pharmaceutical services provided by pharmacists enhance medication adherence and enable the identification and solution of DTPs. To improve people’s pharmaceutical knowledge, drug safety is advocated at community care centers. Pharmacists are tasked with prescription dispensing, medication adherence consultations, and cognitive services. Home or institutional medical care services are provided to specific patients as needed, and the effectiveness of the aforementioned services is monitored through the use of service performance indices and satisfaction surveys. The ultimate goal of this study is to change people’s medication-related attitude and behavior, reduce drug abuse and waste, and increase the prevalence of pharmaceutical services, thereby enabling older adults to receive appropriate medical treatment and establishing Taoyuan City as a city of safe medication and high quality of life.

## 2. Research Design

This study adopted a non-experimental design using a cross-sectional pre and post intervention method.

### 2.1. Method

**Figure 1:**
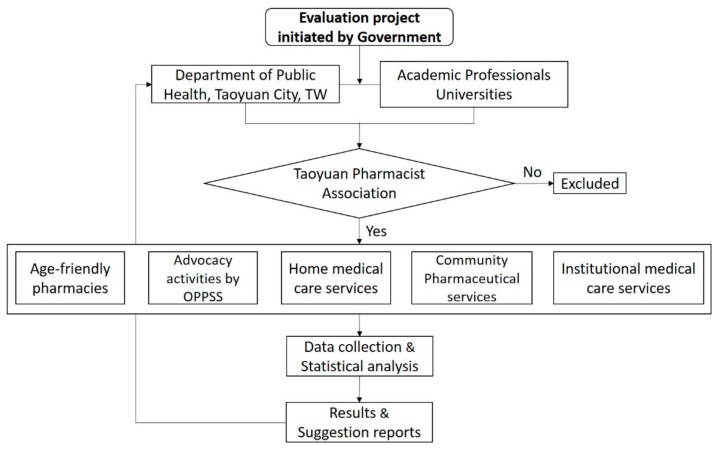
The research process of the study. TW: Taiwan; OPPSS: One Pharmacist Per Service Site.

This study collected data from individuals belonging to the communities in Taoyuan City via a structural questionnaire and 229 pharmacies were involved. From 1 January to 30 November 2018, the Department of Public Health in the Taoyuan City Government collaborated with the Taoyuan Pharmacist Association in enabling pharmacists to provide services in communities, personal residences, and institutions. Indices were established to monitor service performance, and a model was created to collect empirical data for statistical analysis following the guideline of Federation of Pharmacist Association, Taiwan. The inclusion criteria were as follows: (1) Senior citizen above 60 years of age, others were also welcome, (2) capable of communication and understanding the content of questionnaire and (3) having no impairment of consciousness. The Department of Public Health assembled an expert coaching team, which was tasked with establishing age-friendly pharmacies to provide drug safety and free medicine dispensing and cutting services. The team also conducted activities for advocating medicine-related issues which were performed by one pharmacist per service site. Patients failing to abide by the correct medication timing and methods were educated to strengthen their medication awareness. In home medical care, patients’ disease control status was verified, and potential problems in prescriptions by different healthcare institutions or departments were examined. In institutional medication services, correct drug preparation, storage, and delivery were verified, and medical treatment processes were evaluated. The diagram below shows the research process of this study (Figure 1).

### 2.2. Questionnaires

There were two questionnaires adopted for this study. The questionnaire for satisfaction of services consisted of three parts. The first part contained items relating to changes in health behavior and health status. The second part contained items regarding the satisfaction of pharmaceutical services, such as increase in awareness of drug usage, identification of drug side effects, and reduction in waste of medication. The third part of the questionnaire comprised the findings of the survey on public’s view of pharmaceutical services benefits. The second questionnaire for prescription judgement services consisted of nine parts which were modified from Strand LM et al. Drug-Related Problems. [9] TFDA (Taiwan Food and Drug Administration) and Federation of Taiwan Pharmacists Associations have adopted as the SOP (Standard Operation Process) of pharmaceutical services. The code numbers of the prescription judgement services were listed as follows. Code 11–13: The patient requires drug therapy for an indication that would benefit from medications but presently is not being treated with a medication; Code 21–26: the patient does not have a legitimate indication for a medication, which is being taken, and the medication should be discontinued; Code 31–39: the patient is taking a medication that is not able to be effective for the medical condition being treated; Code 41–46: the patient is not taking enough of the medication to be therapeutically effective; Code 51–55: the patient is taking too much of the medication and it is causing a toxic effect; Code 61–67: the patient is experiencing an adverse event as a result of the medication so the medication should be discontinued; Code 71–75: missing dosing from caregiver; Code 81–83: incorrect knowledge from self-care; and Code 91–97: the patient is not able and/or willing to take the medication as intended [10]. In community pharmaceutical services, we adopted the Morisky 8-Item Medication Adherence Questionnaire [11].

### 2.3. Questionaaire Reliability Analysis

A total of four questionnaires for patient satisfaction evaluation were designed to assess the advocacy activities and home, community, and institutional medication services. The questionnaires were pretested for their reliability using internal consistency method. To analyze the reliability of each questionnaire item, the pretest for the advocacy activity, home medical care service, community pharmaceutical service, and institutional medical care service questionnaires was conducted using 30, 28, 37, and 31 interviewees, respectively. The survey results were first entered into SPSS data forms for internal consistency analysis. The Cronbach’s α of the dimensional and overall indices were then calculated through reliability analysis using SPSS 19.0 (IBM Corp., Armonk, NY, USA). The items related to service satisfaction and helpfulness were analyzed. Regarding the dimensional reliability analysis, the original Cronbach’s α, and that after three items had been deleted, were recalculated to determine the overall and item-level reliability and accuracy. The dimensional reliability was improved after these items were adjusted or removed.

#### 2.3.1. Advocacy Activity Questionnaire

A total of 6257 copies of this satisfaction questionnaire were returned for a return rate of 82.3%; 69.2% of the respondents were female, and 52.6% were aged 70 years or older. The average age of the respondents was 65.5 years. The questionnaire consisted of two dimensions: course benefits and arrangement. The overall Cronbach’s α of the questionnaire was 0.977, and the individual Cronbach’s α of course benefits and arrangement was 0.967 and 0.980, respectively; all the values were satisfactory.

#### 2.3.2. Home Care Questionnaire

A total of 295 copies of this satisfaction questionnaire were returned. The questionnaire consisted of two dimensions: service satisfaction and helpfulness. The overall Cronbach’s α of the questionnaire was 0.987, and the individual Cronbach’s α of service satisfaction and helpfulness was 0.967 and 0.988, respectively; all the values were satisfactory.

#### 2.3.3. Community Care Questionnaire

A total of 567 copies of this satisfaction questionnaire were returned. The questionnaire had two dimensions: service satisfaction and helpfulness. The overall Cronbach’s α of the questionnaire was 0.923, and the individual Cronbach’s α of service satisfaction and helpfulness was 0.870 and 0.859, respectively; all the values were satisfactory.

#### 2.3.4. Institutional Medical Care Questionnaire

A total of 75 copies of this satisfaction questionnaire were returned. The questionnaire comprised two dimensions: service satisfaction and helpfulness. The overall Cronbach’s α of the questionnaire was 0.937, and the individual Cronbach’s α of service satisfaction and helpfulness was 0.859 and 0.906, respectively; all the values were satisfactory.

## 3. Results

### 3.1. Age-Friendly Pharmacies

There were 583 NHI-contracted pharmacies in Taoyuan City at the time of this study, and 229 (39.3%) expressed an intention to establish an age-friendly pharmacy environment. Table 1 lists the numbers of customers serviced by the age-friendly pharmacies; the total number of time of customer service were 669,754.

### 3.2. Home Medical Care Services

A total of 40 pharmacists participated in the home medical care services for 293 patients, with 611 visits paid. Of these patients, 50.2% were men; 28.7% were aged 71–80 years; and 78.2% were aged 65 years or older. The average age of the patients was 73.3 years. Of all the patients, 31.1% exhibited no medication problems but were instructed on appropriate medical visit or self-care methods; 12.5% exhibited poor self-care and an unhealthy lifestyle; and 7.2% had improper medication knowledge (Table 2). Regarding the types of suggestions, the pharmacists provided to physicians according to the medication problems of the patients, medication knowledge were suggested regarding 17% of the patients; suggestions on self-care and lifestyles were provided regarding 16.2% of the patients; and those on disease and medical visit knowledge were made for 13.1% of the patients. Regarding the feedback given by the patients on the content of education provided by the pharmacists, 19.2% of the patients expressed that they their lifestyle and self-care skills had consequently improved; 15.3% expressed that their medication knowledge had improved; and 10.7% expressed that their knowledge on diseases and medical visits had improved and that they consequently made fewer clinical visits. A total of 295 copies of the questionnaire for pharmaceutical services satisfaction were returned, and the findings revealed that the home medical care services benefitted the patients substantially and enhanced their drug-safety-related awareness and behaviors. Specifically, 75.2% of the respondents reported that their health improved after they received the home medical care services; 86.4% expressed their intention to increase the time they invested in their health; and 89.1% stated that their attention to their own health had increased after they received the home medical services. Nearly 90% of the respondents were satisfied with the home medical care services. Moreover, more than 90% of the respondents indicated that the home medical care services had improved their knowledge and behaviors relating to medication.

### 3.3. Community Pharmaceutical Services

A total of 636 participants received community pharmaceutical services, 569 of whom received medication adherence consultation and 41 received cognitive services. The pharmacists employed a medication adherence scale to understand the patients’ medication adherence at home; open-ended questions were adopted to enable patients to describe their actual medication conditions at home. The adherence scales (score) were categorized into low (0–6), moderate (7–11) and high (12). The medication adherence consultation services were provided to 569 patients by 31 pharmacists. The total number of types of drugs taken by the patients was 4208, and 2227 of the types were fully administered 585 times. Of the patients, 51.1% were female, and 31.7% were aged 61–70 years. The average age of the patients was 65.8 years, and 69.3% of the patients had two or more chronic diseases (Table 3).

The case analysis on current drug use revealed that the percentage of respondents giving incorrect answers to questions on their actual medication time and dosage was 67.3%; the percentage of respondents correctly answering questions on therapeutic use, dosage form, experience or knowledge of the adverse effects of specific drugs, and forgetting to take medicine or other precautions was 74.5%, 74.5%, 57.2%, and 75.4%, respectively; 20–40% of the patients expressed uncertainty in the questions on drug use, particularly on whether they had experienced or learnt of the adverse effects of specific drugs (41.1%). Table 4 details the patients’ medication adherence before and after the community pharmaceutical service intervention. The number of patients highly cooperative in medication had risen from 14 to 234 after the intervention (a significant increase from 2.4% to 41.3%), and the number with low medication adherence had dropped from 533 to 33 (a significant decrease from 93.7% to 5.8%). This indicated that the patients’ medication adherence was significantly higher after the intervention (Table 4).

The pharmacists provided cognitive services to 41 patients, 61.0% of whom were female; 41.5% of these patients were aged 61–70 years. Their average age was 70.2 years (Table 5).

Of all the medication problems examined, “medication was repeated (the same type or pharmacological classification of drugs)” was the most common among the patients (17.1%) but suggestions were provided on disease control or treatment effectiveness” (14.6%) and “dosage was too high” (14.6%; Table 6). Of all the suggestions provided to the physicians by the pharmacists regarding the medication problems of the patients, suggestions to stop specific medications were the most common (29.3%), followed by suggestions to change the dosage (24.4%) and switch to another medicine (17.1%). Of all the feedback given by the patients regarding the advocacy course provided by the pharmacists, “the physician has not replied for 1 month” had the highest percentage. Of the patients, 26 were referred to five community pharmacists by their physician; 65.4% of these patients were female, and the average age of these patients was 60.8 years. Regarding the medication status of the patients, 80.8% of the patients stated that their actual medication timing or dosage was incorrect; 69.2% and 57.7% indicated that their therapeutic use of medicine and use of dosage forms were correct, respectively; 73.1% reported that they had experienced or learned of the adverse effects of specific drugs; and 42.3% were unsure whether they had forgotten to take medicine and were unsure of other precautions. A total of 567 copies of the questionnaire for patients’ satisfaction were returned. Specifically, 73.6% of the respondents indicated that their health condition had improved substantially, 85.9% that they had increased the amount of time they spent caring for their own health, and 89.1% that they had paid more attention to their own health since receiving the service. Additionally, 97.9% of the respondents were satisfied overall with the age-friendly pharmaceutical service and reported that this service had increased their medication knowledge; 94.2% reported that the service had enabled them to confirm the side effects of specific drugs or factors that affect medication adherence; 94.3% reported that the service had enabled them to confirm the causes of improper medication and learn about, discuss, and correct errors in medication; and 95.7% indicated that the service had improved the curative effects of their prescriptions, reducing the waste of medicine. On the benefits of the service, more than 90% of the respondents reported that the service had improved their medication knowledge and behaviors. Specifically, 97.4% of the respondents indicated that the service had enabled them to understand the correct medication timing and methods; 94.9% indicated that the service had enabled them to confirm the causes of improper medication and learn about, discuss, and correct errors in medication; and 95.7% stated that the service had improved the curative effects of their prescriptions, reducing the waste of medicine (Table 6).

### 3.4. Institutional Medical Care Services

A total of 75 satisfaction questionnaires were returned, and they revealed that 97.3% of the respondents were satisfied with the services. More than 90% of the respondents indicated that the services had significantly improved their medication knowledge and behaviors. Regarding which services the respondents suggested that the pharmacists should provide, 82.7% of the respondents suggested that pharmacists continue to conduct medication evaluations, investigate the appropriateness and safety of medical treatment, and identify, solve, and prevent medication problems; 80.0% hoped that the pharmacists would continue to examine the appropriateness of the medicine prescribed by various medical institutions and departments (Table 7).

## 4. Discussion

This research is a consensus formed by joint discussions between Department of Public Health, Taoyuan City, medical and pharmaceutical experts. The content of the questionnaire had been determined for operability and feasibility in the expert meeting, and each service mode was adapted according to the characteristics of the elderly in Taiwan. The questionnaire is divided into five categories (age-friendly pharmacies, advocacy activities, home medical care services, community pharmaceutical services, and institutional medical care services) according to the current Taiwan pharmaceutical service model. The number of items and contents are based on the guideline from Federation of Pharmacists Association, Taiwan (https://www.taiwan-pharma.org.tw/ (accessed on 4 August 2021)) to fully obtain the opinions of the elderly.

According to their complexity, professional pharmaceutical services are divided into prescription and dispatch services, consultation services, and direct pharmaceutical care [12]. All the services were provided in coordination with the local conditions. Community pharmacies in Taiwan can provide convenient and accessible public health services in accordance with the needs of older adults; thus, the self-care knowledge of older adults was enhanced in this study. The integrated pharmaceutical services, which encompassed community-based, home-based, and institutional medical care services, mitigated the limitations of pharmacy accessibility due to the variability of restrictions in pharmacies [13].

Incorrect medication concept, not understanding the correct time or dosage of medication, wrong dosage or usage, repeated medication (same drug or same pharmacological classification), and complicated administration time of several medications accounted for about 18%. Suffering from two (inclusive) or more chronic diseases, using five (inclusive) or more doctors to dispose of drugs, and having two (inclusive) or more chronic continuous prescription notes, etc., accounted for about 84%. It is speculated that this might correlate with the low adherence in patients. For example, 60% of the elderly in Taiwan suffer from two or more chronic diseases and multiple medications. Because of community pharmaceutical services, the degree of low adherence patients significantly dropped from 533 to 33. It shows that the number of low adherence patients dropped to 5.8%, showing the feasibility and effectiveness of this public health services. In addition, the community pharmacists promoted community-based integrated pharmaceutical services [7], chronic disease management programs, medical treatment management services, and pharmaceutical care performance index monitoring, thereby reducing the number of medical visits made by patients, saving health insurance expenses [14], reducing the number of unsatisfactory medical visits, preventing medication repetition, and improving the self-medication behaviors of older adults as well as medication safety. The quality of medical care would be markedly improved [15].

## 5. Conclusions

Age-friendly pharmacies provide services in accordance with the needs of local residents. Each administrative district has a local population with unique health needs, thus requiring a specific pharmaceutical service model [16]. The feasibility of the incorporation of integrated public health services into age-friendly pharmacies was confirmed by this study. Improvements were made to the unsatisfactory aspects of the services to enhance people’s service satisfaction. Funds were invested, and laws and regulations were reviewed to supplement the service items that could not be provided through the NHI, such as transportation, thus establishing a business model in line with the existing public healthcare situation and reinforcing sustainable healthcare development [17]. The public health services and age-friendly pharmacies involves multi-faceted interaction; we take public views into consideration and as suggestions for the priority order of governance. Therefore, at the present stage, categorical variables are presented in a ratio to assess the practical feasibility and the reference for follow-up policies. The SEM method and McDonald’s omega statistical analysis will be adopted in future research and compared to Cornbach α coefficient. In home care pharmaceutical services, the main problem lies in patients’ incorrect knowledge of drugs and poor self-care. Through the services of the government and pharmaceutical professionals, it has been shown that more than 90% of the people believe in-home care pharmaceutical services were helpful to improve the cognition and behavior related to medication. In terms of community pharmaceutical services, community pharmacist sites provide consultation services on medication adherence. The medication adherence of patients has improved significantly. The number with low medication adherence had dropped, showing that community pharmaceutical services can indeed improve people’s knowledge of medication and deepen their understanding of medication indications and interactions. Regarding institutional medical care services, the evaluation of residents’ drug treatments and the discussion on the appropriateness and safety show that more than 90% of the people believe that pharmacists’ visits to institutional roving services are of great help to the appropriateness and safety of their medication.

Future studies will focus on building integrated care models that involve physicians referring patients to pharmacists before discharge to further implement interdisciplinary cooperation [14]. In coordination with the development of Taoyuan City as a smart city, the Internet of Things and artificial intelligence will be applied to create a care model that satisfies cost–benefit relationships and the needs of elderly community residents, thereby attaining a network of community pharmacy support [18].

## Figures and Tables

**Table 1:** Number of times of customer service provided by age-friendly pharmacies.

Service	Customers Serviced
Medication guidance, consultation, and instructions	347,217
Blood pressure measurement	90,149
Referral services (for healthcare services related to diabetes care, the second-generation smoking cessation program, and long-term care)	34,750
Provision of reading or magnifying glasses to older adults	32,648
Guidance on discarding expired drugs	98,501
Guidance on operating blood pressure and glucose monitors	66,489

**Table 2:** Medication problems discovered by the pharmacists in the home medical care services (*N* = 582).

Code *	Content of Communication	No. of Times	Percentage (%)
0	No medication problem was discovered, but the patient was educated on appropriate medical visit and self-care methods	181	31.1%
82	The patient exhibited poor self-care and an unhealthy lifestyle	73	12.5%
92	Medication knowledge was incorrect	42	7.2%
94	The patient frequently forgot to take medicine	30	5.2%
93	The patient did not understand the correct medication timing or dosage	29	5.0%
81	Knowledge on diseases and medical visits was incorrect	25	4.3%
96	Physiological self-monitoring was required	21	3.6%
71	Medicine was not administered	19	3.3%
01	(To physicians) No medical treatment was applied, but suggestions were provided on disease control or treatment effectiveness tracking	16	2.7%
73	Dosage or administration method was incorrect	16	2.7%
83	The patient’s knowledge on over-the-counter drugs, health food, or Chinese medicine was incorrect	16	2.7%
02	The patient was referred to personnel in other professional fields	15	2.6%
98	The patient did not understand the use of the dosage form	13	2.2%
22	Medication was repeated (the same type or pharmacological classification of drugs)	10	1.7%
97	Drug storage was improper	9	1.5%
31	Dosage form was improper	8	1.4%
11	Untreated conditions or diseases were discovered	7	1.2%
91	Administration time of several drugs was excessively complex	6	1.0%
25	Test data for medicine were lacking	5	0.9%
32	Treatment contraindications were identified	5	0.9%
33	Drug incompatibility was identified	5	0.9%
13	Combined usage with other drugs was required to reinforce the treatment	4	0.7%
35	More effective, safer, more convenient, or cheaper drugs were available	4	0.7%
65	Unexpected pharmacological reactions were identified under normal dosage	4	0.7%
38	This medicine had proven to be ineffective in its previous prescriptions	3	0.5%
55	The patient exhibited poor liver and kidney functions	2	0.3%
64	The prescription was unsafe for the patient (e.g., the patient had risk factors for diseases, was pregnant, was breastfeeding, was a toddler, or was elderly)	2	0.3%
74	Incorrect administration time was given	2	0.3%
95	The patient was unable to swallow the medicine	2	0.3%
12	Preventive medical treatment was required	1	0.2%
21	No indication was discovered from this drug	1	0.2%
36	Medication timing was excessively complicated	1	0.2%
37	Single-ingredient medicine as recommended over compound medicine	1	0.2%
51	Dosage was too high	1	0.2%
61	Drug interactions were identified	1	0.2%
67	Drug administration was incorrect	1	0.2%
75	Drug administration was too fast	1	0.2%

* Prescription judgement services code [10].

**Table 3:** Inclusion criteria of the patients receiving medication adherence consultations (*N* = 522).

Variable	No. of Patients	Percentage (%)
Inclusion criteria (missing value: *n* = 4)		
Diagnosed with two or more chronic diseases	359	69.3%
Using five or more types of medicine prescribed by physicians	78	15.1%
Having two or more continuous prescriptions for chronic diseases	73	14.1%
Using a special dosage form	8	1.5%
Sex (missing value: *n* = 5)		
Female	264	51.1%
Male	253	48.9%
Male-to-female ratio	1.04	
Age (years; missing value: *n* = 4)		
<40	17	3.3%
41–50	45	8.7%
51–60	107	20.7%
61–70	164	31.7%
71–80	110	21.2%
81–90	63	12.2%
≥91	12	2.3%
Mean ± standard deviation (years)	65.8 ± 13.4	

**Table 4:** Patients’ medication adherence (*N* = 569).

Medication Adherence	Adherence Score	Before Intervention		After Intervention	
		No. of Patients	Percentage (%)	No. of Patients	Percentage (%)
Low	0	11	1.9%	0	0.0%
	1	2	0.4%	0	0.0%
	2	38	6.7%	0	0.0%
	3	61	10.8%	6	1.1%
	4	97	17.1%	8	1.4%
	5	110	19.4%	8	1.4%
	6	214	37.7%	11	1.9%
Moderate	7	6	1.1%	42	7.4%
	8	3	0.5%	53	9.3%
	9	2	0.4%	65	11.5%
	10	5	0.9%	69	12.2%
	11	4	0.7%	71	12.5%
High	12	14	2.4%	234	41.3%
Missing value		2	-	2	-

**Table 7:** Institutional medical service needs (*n* = 75).

Service Item	No. of Respondents	Percentage (%)
Confirm the appropriateness of drug preparation, storage, and delivery.	46	61.3
Confirm the appropriateness of combined administration of prescribed medicine with other types of medicine, such as Chinese medicine and health food.	54	72.0
Examine the appropriateness of the medicine prescribed by various medical institutions and departments.	60	80.0
Conduct medication evaluation, investigate the appropriateness and safety of medical treatment, and identify, solve, and prevent medication problems.	62	82.7
Provide drug information consultation, quality control services, and on-the-job education to patients and medical professionals.	53	70.7

**Table 5:** Demographic information of the patients receiving cognitive services (*N* = 41).

Variable	No. of Patients	Percentage (%)
Inclusion criteria		
Diagnosed with two or more chronic diseases	18	43.9%
Using five or more types of medicine prescribed by physicians	6	14.6%
Having two or more continuous prescriptions for chronic diseases	7	17.1%
Using a special dosage form	1	2.4%
Referred by a physician	9	22.0%
Sex		
Female	25	61.0%
Male	16	39.0%
Male-to-female ratio	1.6	
Age (years)		
<40	1	2.4%
51–60	6	14.6%
61–70	17	41.5%
71–80	7	17.1%
81–90	9	22.0%
91≥	1	2.4%
Mean ± standard deviation (years)	70.2 ± 11.7	

**Table 6:** Medication problems identified by the pharmacists (*N* = 41).

Content of Communication	No. of Times	Percentage (%)
Medication was repeated (the same type or pharmacological classification of drugs)	7	17.1%
(To physicians) No medical treatment was applied, but suggestions were provided on disease control or treatment effectiveness tracking	6	14.6%
Dosage was too high	6	14.6%
Dosage form was improper	4	9.8%
Unexpected pharmacological reactions were identified under normal dosage	4	9.8%
No indication was discovered from this drug	3	7.3%
The patient exhibited poor liver and kidney functions	2	4.9%
Untreated conditions or diseases were discovered	1	2.4%
Another drug could be administered to prevent the side effect	1	2.4%
More effective, safer, more convenient, or cheaper drugs were available	1	2.4%
Single-ingredient medicine was recommended over compound medicine	1	2.4%
Dosage or serum drug concentration was too low	1	2.4%
Dosing interval was too long	1	2.4%
The patient was allergic to this drug	1	2.4%
The prescription was unsafe for the patient (e.g., the patient had risk factors for diseases, was pregnant, was breastfeeding, was a toddler, or was elderly)	1	2.4%
Dosage or administration method was incorrect	1	2.4%

## Data Availability

The data of this study are available from the corresponding authors upon request.
